# A block staining method using ethanolic phosphotungstic acid for the visualisation of collagens in transmission electron microscopy

**DOI:** 10.1371/journal.pone.0339342

**Published:** 2026-02-10

**Authors:** Astrid Obermayer, Stefan Hainzl, Ulrich Koller, David Lang, Bernd Lamprecht, Walter Stoiber

**Affiliations:** 1 EM Core Facility, Department of Environment and Biodiversity, University of Salzburg, Salzburg, Austria; 2 EB House Austria, Research Program for Molecular Therapy of Genodermatoses, Department of Dermatology and Allergology, University Hospital of the Paracelsus Medical University Salzburg, Salzburg, Austria; 3 Department of Pulmonology, Kepler University Hospital, Johannes Kepler University Linz, Linz, Austria; Advanced Materials Technology Research Institute, National Research Centre, EGYPT

## Abstract

Conventional transmission electron microscopic imaging of biological samples requires contrast enhancement by staining with heavy metal salts. The most widely used stains are uranyl acetate and its non-radioactive lanthanoid replacements, and lead citrate. However, these substances proved of limited use for the visualisation of small fibrillar collagens. We therefore developed a preparation of ethanolic phosphotungstic acid (E-PTA) as an improvement to overcome this deficiency. We were able to establish a highly effective and time saving block staining procedure that can be integrated in the dehydration steps. The method reliably visualizes fibrillar collagens, prominently including the small collagen VII anchoring fibrils of the human skin, and various other extracellular matrix components. E-PTA-stained collagen I/III fibrils are conspicuous in transverse and longitudinal section, accurately showing the characteristic banding pattern in the latter. The new E-PTA based block staining method also clearly depicts all relevant intracellular structures, particularly accentuating keratin fibres and desmosomal and hemidesmosomal plaques. We therefore conclude that beyond the visualization of collagen, this method is also a fast, inexpensive and versatile non-radioactive alternative to standard staining methods.

## Introduction

In various biological and biomedical fields there is an increasing demand for high resolution imaging to analyze the structures, positional relationships and interactions of subcellular components. In many cases, the method of choice is transmission electron microscopy (TEM), and although more advanced techniques such as cryo-TEM are of increasing interest, many users continue to prefer conventional TEM on ultrathin resin sections, be it due to higher availability, lower cost or higher throughput rates.

In conventional TEM of biological materials, it is necessary to introduce large-atomic-mass elements to enhance scattering contrast. This is commonly achieved by introducing heavy metal salts. The most widely used stains in this context are aqueous or ethanolic solutions of uranyl acetate (UA) and lead citrate [1,2]. In negative staining of small particulate specimens mounted on girds, these salts are adsorbed to the carrier, creating a stained halo around the largely unstained target structures. The more frequently applied positive staining is achieved by attaching the heavy metal salts directly to the structures to be visualized. This is done either by specimen infiltration prior to resin embedding and sectioning (pre-embedding or block staining procedure), or by incubation of ultrathin sections or particulate on-grid specimen (post-embedding on-grid procedures). UA has been most effectively used over decades on a multitude of cell types and tissues in all variants of TEM staining but is now increasingly banned from TEM due to safety regulations on nuclear materials [3,4]. Lanthanoid salts have been successfully introduced as non-radioactive replacements for UA in many of the routine applications, including the widely used combination with lead citrate [4,5].

However, depending on specimen type and the physical and chemical circumstances of sampling, these routine procedures have proven limited in their potential to visualize specific micro-structural targets, prominently including fibrous collagens. Key examples from our own research that induced us to improve collagen visualization in TEM investigation are the collagen VII anchoring fibrils of the human skin (extensive preliminary analyses for [6]), and the collagenous formations of lung tissue from patients with pulmonary fibrosis sampled by transbronchial cryobiopsy [7]. Both kinds of specimens present a diagnostic need for optimized fine structural imaging of collagens in their specific micro-environment.

Collagen VII anchoring fibrils are critical for a stable attachment of the epidermis to the underlying dermis, and therefore subject of study in research on skin-affecting conditions such as epidermolysis bullosa (EB) [8,9]. Their structure and exact position in the hemidesmosome contacts along the dermal-epidermal junction [10] can only be visualized by electron microscopy. Such imaging is an essential control required in current approaches to develop gene regulation-based EB therapies using in vitro models (e.g., [6]), but routine methods of section staining proved insufficient to generate adequate contrast.

A similar situation applies to the analysis of collagen structure change in the fibroblastic foci of lung parenchyma in interstitial lung diseases (ILD). The dynamics of fibrillar collagen synthesis and turnover, in particular concerning collagens I and III, have been identified as crucial for the progression of the diseases [11,12]. Since its introduction in the late 2000s, transbronchial cryobiopsy has widely replaced previous riskier or more tissue damaging methods for lung parenchyma sampling in ILD. Tissue specimens harvested by this technique have proved to be of sufficient size and quality for the purposes of paraffin-based light microscopic pathology [13,14]. However, when using TEM, improvement of the present staining technology turned out important to allow for effective fine structural analyses, and for better judgement of the micro-artifacts induced by the unavoidable freeze-thaw cycle during tissue extraction.

To address these demands, we developed a new staining method based on the proven role of heteropolyacids (phosphomolybdic acid and phosphotungstic acid) in the selective visualization of collagens in paraffin section staining using trichrome methods or hematoxylin [15,16]. Phosphotungstic acid H_3_[PW_12_O_40_] (PTA) is a complexly structured hexahydrate compound that assembles 12 tungsten atoms octahedrally linked by oxygen atoms around a central phosphorous atom [17]. This special combination of a non-metal (phosphorus) and an electron-dense metal (tungsten) of high atomic mass (amu 183.5) is known to interact with positively charged proteins. Despite its wide use in procedures of collagenous connective tissue staining for light microscopy and a niche application in TEM negative staining [18,19], only a few sources have reported on the use of PTA in pre- or post-embedding positive TEM staining [3,20–22], with even fewer making use of this substance’s particular affinity to collagens in corresponding analyses [23,24].

We tested a preparation of ethanolic PTA (E-PTA) adapted from a protocol originally used to stain retinal and synaptic structures [22] for its suitability to improve collagen visualization in TEM analysis of the specimen types defined above. We were able to establish a highly effective, non-radioactive block staining procedure integrable into the dehydration process that can be efficiently used as a stand-alone method of contrast enhancement. Fibrillar collagens including collagen VII anchoring fibrils are reliably visualized, together with all other relevant fine structural details, with no need for additional post-embedding stains.

## Materials and methods

The protocol described in this peer-reviewed article is published on https://www.protocols.io, DOI: https://doi.org/10.17504/protocols.io.rm7vz9m9xgx1/v1 and is included for printing as supporting information S1 File with this article.

### Ethics declarations

All procedures described in this study are in full accordance with Austrian legislation and with the Declaration of Helsinki.

Human skin samples and skin equivalents were obtained upon written informed consent in the course of a larger study (see Ref. [6]) at the Department of Dermatology and Allergology of the University Hospital Salzburg, Austria. Ethical approval was granted by the ethics committee of the County of Salzburg (vote numbers: 415-E/2118/34–2021 [skin samples], 415-E/2118/45–2024 [skin equivalents]).

Human lung tissue samples were obtained upon written informed consent in the course of a larger study on patients in clinical evaluation of ILD (see ref. [7]) at the Department of Internal Medicine 4 – Pneumology of the Kepler University Hospital in Linz, Austria. Ethical approval was granted by the ethics committee of the medical faculty of the Johannes Kepler University in Linz (vote number: EK Nr. 1287/2022).

## Expected results

We tested the presented protocol of E-PTA block staining on samples of murine and human skin and in-vitro skin equivalents, and of human lung parenchyma derived from cryobiopsies. Our results show that the method is highly suitable for depicting collagens and other extracellular matrix components, while also generating adequate contrast throughout all relevant cellular structures.

**Fig 1:**
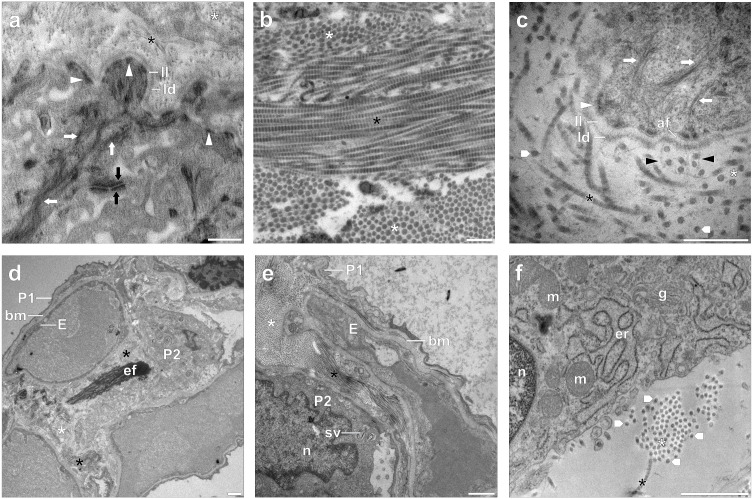
PTA block staining of human and murine skin tissue and human lung parenchyma. Labelling. White asterisks: transversal sections of collagen I/III fibrils, black asterisks: longitudinal sections of collagen I/III fibrils, white arrows: keratin fibres, black arrows: desmosomes, white arrowheads: hemidesmosomes, black arrowheads: collagen VII anchoring fibrils, white pentagon arrows: collagen I/II fibril profiles showing inhomogeneous staining patterns. af: anchoring filaments bm: basal membrane, E: endothelial cell, ef: elastic fibres, er: endoplasmic reticulum, g: Golgi apparatus, ld: lamina densa, ll: lamina lucida, m: mitochondrion, n: nucleus, P1: type 1 pneumocyte, P2: type 2 pneumocyte, sv: surfactant vesicle. **(a)** Healthy human skin, overview of the dermoepidermal junction. PTA prominently stains the keratin fibres and desmosomal and hemidesmosomal plaques of the keratinocytes. Aggregates of collagen I/III fibrils and the lamina lucida and lamina densa sublayers are clearly visible. **(b)** Healthy murine dermis. Collagen I/III fibrils are stained at high contrast, with clearly visible banding in longitudinal stretches. **(c)** Human in-vitro skin equivalent of a healthy control donor, dermoepidermal junction. E-PTA-staining accentuates the keratin fibres connecting to the hemidesmosomes. Anchoring filaments traversing the lamina lucida and collagen VII anchoring fibrils connecting the basal lamina to the dermal collagen are clearly visible. Again, the banding of longitudinally sectioned collagen I/III fibrils is well defined. Transversal profiles of these fibrils frequently show inhomogeneous staining patterns. (d,e,f) Fibrotic human lung parenchyma. **(d)** Low magnification overview. E-PTA staining allows for the differentiation of all cellular and extracellular components and particularly accentuates the elastic fibres. **(e)** Detail of an alveolar septum. Large deposits of collagen are clearly visible, pneumocytes and endothelial cells are well defined. **(f)** Detail of an alveolar fibroblast demonstrating the suitability of E-PTA for staining of intracellular components. Mitochondria, Golgi apparatus and endoplasmic reticulum are clearly defined, ribosomes and nuclear chromatin are especially well stained. Scale bars: (a-c): 0.5 µm, (d-f): 1.0 µm.

**Fig 2:**
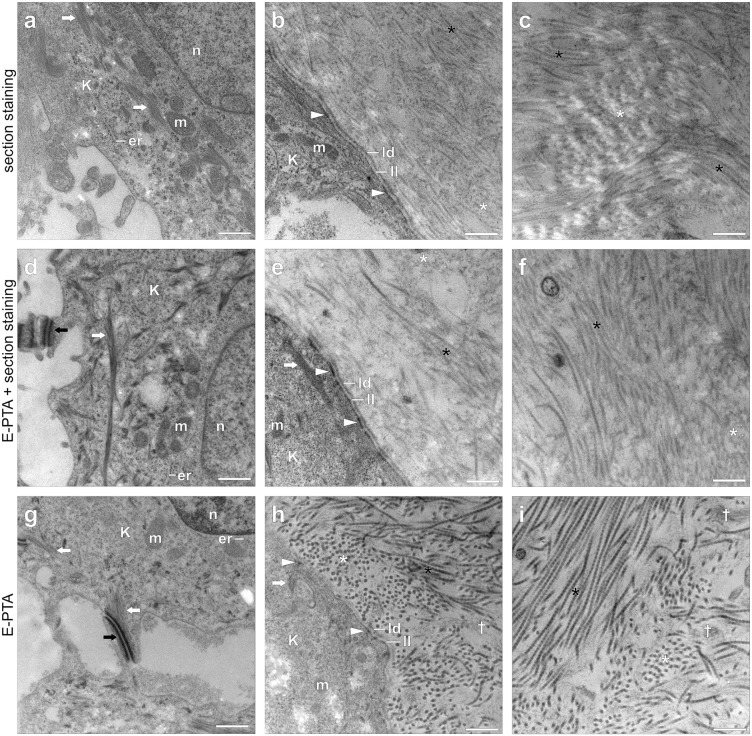
Comparison of staining methods on human skin equivalents. **(a-c)**: Section staining using UA replacement stain and lead citrate (alternative 1). **(d-f)**: Combination of E-PTA and sections staining as above (alternative 2). **(g-i)**: E-PTA block staining. Labelling. White asterisks: transversal sections of collagen I/III fibrils, black asterisks: longitudinal sections of collagen I/III fibrils, white arrow: keratin fibres, black arrows: desmosomes, white arrowheads: hemidesmosomes, white daggers: elastic fibrils. er = endoplasmic reticulum, K = keratinocyte, ld: lamina densa, ll: lamina lucida, m = mitochondrion, n = nucleus. Intracellular components are well defined with all staining methods (a,d,g). Ribosomes and inner membrane structures are slightly better visible with routine section staining alone (a,b), although the combination of E-PTA and section staining results in the best overall intracellular contrast (d,e). Keratine fibres are strongly stained by E-PTA (g,h), and this effect is further enhanced by additional section staining (d,e). The basal lamina at the dermoepidermal junction is visible with all staining methods (c,f,i). The potential of PTA block staining is best demonstrated by its effects on desmosomes (d,g) and hemidesmosomes (e,h), and by its superior staining of collagen fibrils. Routine section staining results in poor definition of collagen I/III fibrils, especially in cross sectional view (b,c). By contrast, E-PTA stained collagen I/III fibrils always exhibit high contrast and a clearly visible banding pattern (h,i). Additional section staining weakens E-PTA collagen contrast rather than improving it (e,f). Scale bars: 0.5 µm.

In murine and human skin and in-vitro skin equivalents, the method reliably visualizes collagen VII anchoring fibrils together with their micro-morphological environment at the dermal-epidermal junction. Hemidesmosomal plaques connecting to strongly stained keratin fibres and the epidermal basal lamina are clearly displayed. The sublayers of the basal lamina, the lamina lucida and lamina densa, are well discernible. The lamina densa at the dermal border which contains collagen IV and embeds the endings of the collagen VII anchoring fibrils is visualized in high detail (Fig 1a-1c). This allows reliable analysis of EB skin samples in which these structures are defective (S1 Fig) and functionality testing of organoid models generated to enable genetic repair of anchoring fibril formation [6]. Similar to the above structures, the laminin 332 (formerly laminin-5) containing anchoring filaments [25] that traverse the lamina lucida to connect the epidermal hemidesmosomes with the lamina densa are clearly displayed (Fig 1a-1c). The elastic fibrils of the human dermis are depicted with similar quality (Fig 2h, 2i) [26].

The more prominent collagen I/III fibrils of the dermis are clearly visualized at all angles of intersection. Longitudinally sectioned stretches of these fibrils show the characteristic 67 nm banding pattern [27,28] at high contrast (Fig 1b, 1c). Profiles of transverse sections frequently show irregular patterns of darker dotted or threadlike structures arranged within a lighter stained matrix (Figs 1c, 1f). This may reflect the different inter- and intrasubfibrillar binding intensities of PTA to the axial packing arrangement of the right-handed triple helix collagen molecule in the quaternary structure of aldehyde fixed collagen fibrils [29]. These fine structure details are rarely discernible when collagen I/III fibrils are stained with other techniques (cf. [30]).

The E-PTA method proved similarly successful in visualizing fibrillar type I/III collagen in the lung parenchyma at all sectioning planes, together with many other interstitial matrix components (Fig 1d-1f, see also [7]). Again, collagen fibrils sectioned at longitudinal and oblique orientation exhibit a pronounced high-contrast banding pattern (Fig 1f). The elastic fibres of the alveolar wall stain conspicuously dark and mostly homogeneous but are sometimes also interspersed with areas of even higher electron density (Fig 1d, 1e). The relative positions and arrangements of all fibrillar and fibrous components are always clearly discernible (e.g., Fig 1d, 1e).

E-PTA staining also provides a very clear definition of the biomembranes of cells and organelles, thus enabling a reliable detection and interpretation of cryo-induced artifacts such as membrane ruptures and mitochondrial and endoplasmic reticulum swelling (Fig 1f).

To further validate our new approach, we compared the results of E-PTA block staining applied as single method with those of conventional section staining with UA replacement stain and lead citrate (alternative 1), and those of a triple combination of E-PTA block staining and conventional section staining with UA replacement stain and lead citrate (alternative 2).

Results proved conclusive: The basal laminae at the dermo-epidermal-junction and of the capillary endothelia in the lung are sufficiently defined with all methods. The same holds for all relevant intracellular structures, although with distinct differences. ER membranes and mitochondrial cristae appear somewhat more distinct with conventional section staining (alternative 1) (Fig 2a, 2b), while the combined method (alternative 2) seems to result in the best overall intracellular contrast (Fig 2d, 2e). Epidermal keratin fibres are strongly stained by E-PTA alone, but this effect is further enhanced with the combined method (alternative 2). A clear superiority of E-PTA block staining becomes evident with desmosomes and hemidesmosomes, and most importantly with fibrillar collagens (Fig 2h, 2i). Conventional heavy metal section staining (alternative 1) is insufficient to clearly visualize collagen VII anchoring fibrils, and often also results in limited or poor definition of collagen I/III fibrils, even more so in cross sectional and nearly cross-sectional views (Fig 2b, 2c). By contrast, E-PTA block staining always defines these elements with high contrast and in the case of collagen I/III with a clearly visible banding pattern (Fig 1b, 1c, 1f and 2h, 2i). The combined procedure of E-PTA and conventional section staining (alternative 2) deteriorates the PTA collagen contrast rather than to improve it (Fig 2e, 2f).

## Conclusions

Our results show that block staining with ethanolic phosphotungstic acid (E-PTA) is a reliable stand-alone staining method for TEM analysis of biological materials from the tissue level down to subcellular scales in the nanometer range. The method proves particularly effective to visualize fibrillar collagens including collagen VII anchoring fibrils while also providing good contrast of all further inter- and intracellular structures. Keratin fibres and desmosomal and hemidesmosomal plaques are particularly accentuated. This may expand the methodological scope of research on a much wider field of collagen-affecting genetic and/or degenerative skin disorders (e.g., [31–33]), also encompassing wound healing, photodamage and age-related change [34,35]. To date, the expanding research in these fields has only rarely employed TEM, and if so, routine heavy metal stains were used [32,35]. Likewise, the method could also fruitfully add to the current research on the role of collagens in tumor biology [36–38].

In summary, this staining method is a full-fledged fast non-radioactive alternative to originally uranyl acetate-based methods of pre- and post-embedding positive staining, with the added advantage that the required reagents are of comparatively low cost. Accordingly, this E-PTA protocol may be of relevance to various types of fine structure research including volume EM techniques.

## Supporting information

S1 File Step-by-step protocol, also available on protocols.io. (DOCX)

S1 Fig E-PTA staining of EB skin. Dermoepithelial junction as present in samples of human-skin-equivalent organoids generated from donor cells of patients with recessive dystrophic epidermolysis bullosa (RDEB). Labelling. White asterisks: transversally sectioned collagen I/III fibrils in extracellular matrix, white arrows: keratin fibres, white arrowheads: hemidesmosomes, E: endothelial cell, ld and ll: lamina densa and lamina lucida of basement membrane, respectively, m: mitochondrion. (a) E-PTA staining accentuates keratin fibres inside the epidermal cells and hemidesmosomes connecting to an incompletely developed basal lamina. As characteristic for RDEB, collagen VII anchoring fibrils are missing, and no collagen I/III fibrils can be found in the vicinity of the epithelial cell. (b) Motif similar to that of (a) but showing a more conspicuous basal lamina, with the lamina lucida and lamina densa sublayers being clearly visible at the site of the hemidesmosome. Again, no collagen VII anchoring fibrils are discernible, and only a few collagen I/III fibrils are present in the matrix close to the cells.
(TIF)
