# Supplementary material for: A block staining method using ethanolic phosphotungstic acid for the visualisation of collagens in transmission electron microscopy
Source: PLoS One. 2026 Feb 10;21(2):e0339342. doi: 10.1371/journal.pone.0339342 (PMC12890093; doi:10.1371/journal.pone.0339342)
Supplement: S1 File — (DOCX) [file pone.0339342.s001.docx]

# S1: Step-by-step protocol, also available on protocols.io

## Guidelines

This protocol describes the workflow of animal and human tissue sample fixation with aldehydes and osmium tetroxide, followed by an innovative block staining procedure with ethanolic PTA integrated into the dehydration steps, and subsequent epoxy resin embedding. Although originally aimed at the visualization of collagen fibrils, the block staining procedure turned out a much more versatile method. Accordingly, it is possible to adapt fixation steps or incubation times to the specific requirements of the sample and/or the established procedures of your lab.

The materials section provides a suggested list of chemicals, but the exact lots and vendors of the components listed are not critical. However, it should be made sure that non-denatured ethanol and EM-grade fixatives are used.

If a resin different to the one listed is used, infiltration times must be adapted according to manufacturer’s instructions.

Cacodylate buffer can be substituted with PBS, but it should be kept in mind that the fixation quality of structures such as intracellular membranes benefits from cacodylate buffer containing calcium ions.

It is best to use glassware as specimen vials, as glass is resistant to propylene-oxide. If you prefer to use plastic ware, please test its suitability beforehand.

Choose reagent volumes according to sample size and use at least 20x the sample volume to ensure that the sample is fully immersed in and surrounded by the liquid.

## Before start

This protocol comprises working steps for 4 lab days, so make sure you plan them accordingly before you start. As some process step times can be altered depending on sample type and other requirements, the number of required days may change.

After the last washing step following fixation with OsO_4_, samples may be interim stored in 70% ethanol at 4°C overnight or for up to approximately 2 weeks, e.g. enabling to collect samples from several experiments. Caution: Do not store in higher concentrations of ethanol, as this might lead to severe shrinking artefacts. Alternatively, interim storage in buffer is also possible, however, this does not provide against microbial contamination as the washing steps are usually not performed under sterile conditions.

## Warnings

Perform all steps under a fume hood and wear protective gear.

Osmium tetroxide is highly reactive, severely toxic and corrosive. Handle with extreme care, avoid any contact with the solution or the fumes. Collect all solutions and the buffer of at least the first two washing steps for special waste disposal according to manufacturer’s instructions and local rules.

Glutaraldehyde and paraformaldehyde are toxic, corrosive and environmental hazards. Avoid any contact with the solutions and the fumes. Collect all solutions and the buffer of at least the first two washing steps for special waste disposal according to manufacturer’s instructions and local rules.

Cacodylic acid is toxic and an environmental hazard. Avoid any contact with the solution and the fumes. Collect all solutions for special waste disposal according to manufacturer’s instructions and local rules.

## Materials

- Dimethylarsinic acid sodium salt trihydrate, molecular weight: 214.03 g/mol, CAS-nr: 6131-99-3, EG-nr.: 204-708-2, Carl Roth 5169.1
- Osmium tetroxide, (4% solution), molecular weight: 254.23 g/mol, CAS-nr: 20816-12-0, EG-nr. 244-058-7, Sigma-Aldrich 251755
- EM-grade glutaraldehyde (1,5 pentanedial) 25 % aqueous solution, molecular weight: 100.12 g/mol, CAS-nr: 111-30-8, EG-nr. 203-856-5, Carl Roth 4157.1
- Paraformaldehyde, molecular weight: 30,03 g/mol, CAS-nr: 30525-89-4, EG-nr. 608-494-5, Carl Roth 0964.1
- Calcium chloride, molecular weight: 110.98 g/mol, CAS-nr: 10043-52-4, EG-nr.233-140-8, Sigma-Aldrich C5670
- Phosphotungstic acid, molecular weight: 2880,17 + x H2O g/mol, CAS-nr: 12501-23-4, EG-nr. 603-020-3, Carl Roth 2635.1
- 96% Ethanol undenatured, molecular weight 46,07 g/mol, CAS nr. 64-17-5, EG-nr. 200-578-6, Carl Roth P075.4
- 100% Ethanol undenatured, molecular weight 46,07 g/mol CAS nr. 64-17-5 EG-nr. 200-578-6, Carl Roth 9065.4
- 1,2-Propylene-oxide, molecular weight: 58.08 g/mol, CAS-nr. 75-56-9, EG-nr. 200-879-2, Sigma Aldrich 8.07027
- Agar Low Viscosity Resin (ALVR) Kit, Agar Scientific, AGR1078

**Solutions:**

**10% PFA stock solution** (100 ml)

10 g paraformaldehyde

Fill to 100 ml with aqua bidest, dissolve by heating to approx. 60°C (do not boil!) and adjust pH with NaOH until the solution appears clear.

Store at 4C° up to one month, or at -20°C for several months.

**CCB buffer** (100 mM cacodylate buffer containing 2mM CaCl_2_) (1L)

21,403 g dimethylarsinic acid sodium salt trihydrate (M=214,03g/mol) and

0,222 g CaCl_2_ (M=110,98g/mol)

Fill to1L with aqua bidest. and stir until fully dissolved.

Adjust pH to 7.4

**2% PFA, 2.5% GA fixative** (50 ml)

10 ml 10% PFA stock solution

5 ml 25% glutaraldehyde aqueous solution, EM grade

35 ml CCB

Best prepared fresh before use, store at 4°C up to one month

**1% OsO_4_ solution** (10 ml)

2.5 ml 4% OsO_4_ solution

7.5 ml CCB

Prepare fresh immediately before use. Very toxic, handle with care!

**Ethanol dilutions** (96ml each)

70% ethanol: 70 ml 96% ethanol, fill to 96 ml with aqua bidest. (26 ml).

80% ethanol: 80 ml 96% ethanol, fill to 96 ml with aqua bidest. (16 ml).

90% ethanol: 90 ml 96% ethanol, fill to 96 ml with aqua bidest. (6 ml).

**1% ethanolic phosphotungstic acid** (10 ml)

0.1 g phosphotungstic acid

Fill to 10 ml with 70% ethanol

Prepare fresh immediately before use

## Steps

1. Primary fixation

- Place samples in a solution of 2% Paraformaldehyde and 2.5% Glutaraldehyde in 100mM cacodylate buffer containing 2mM CaCl_2_ (CCB) 4°C over night
- Wash in CCB, room temperature (RT) 15 min (1/4)
- Wash in CCB, RT, 15 min (2/4)
- Wash in CCB, RT, 15 min (3/4)
- Wash in CCB, RT, 15 min (4/4)

1. Secondary fixation
   - Fix samples in a freshly prepared solution of 1% OsO4 in CCB RT 3h

- Wash in CCB, RT, 15 min (1/4)
- Wash in CCB, RT, 15 min (2/4)
- Wash in CCB, RT, 15 min (3/4)
- Wash in CCB, RT, 15 min (4/4)
  - If applicable store samples in 70% ethanol at 4°C over night or until further use (up to approx. 2 weeks).

1. Block staining
   - Incubate samples in a freshly prepared solution of 1% PTA in 70% ethanol, RT, 1h
2. Dehydration
   - 80% ethanol, RT, 30 min
   - 90% ethanol, RT, 30 min
   - 96% ethanol, RT, 30 min
   - 100% ethanol, RT, 15 min (1/3)
   - 100% ethanol, RT, 15 min (2/3)
   - 100% ethanol, RT, 15 min (3/3)
3. Infiltration
   - Propylenoxide, RT, 15 min (1/3)
   - Propylenoxide, RT, 15 min (1/3)
   - Propylenoxide, RT, 15 min (1/3)
   - 1:1 mixture of propylenoxide:ALVR resin, RT 1h
   - 1:2 mixture of propylenoxide:ALVR resin, RT, 2h
   - ALVR resin, RT, over night
4. Embedding
   - Place in fresh resin in suitable embedding moulds
   - Cure, 60°C, 24 h
