# Supplementary figures and images for: A block staining method using ethanolic phosphotungstic acid for the visualisation of collagens in transmission electron microscopy
Source: PLoS One. 2026 Feb 10;21(2):e0339342. doi: 10.1371/journal.pone.0339342 (PMC12890093; doi:10.1371/journal.pone.0339342)

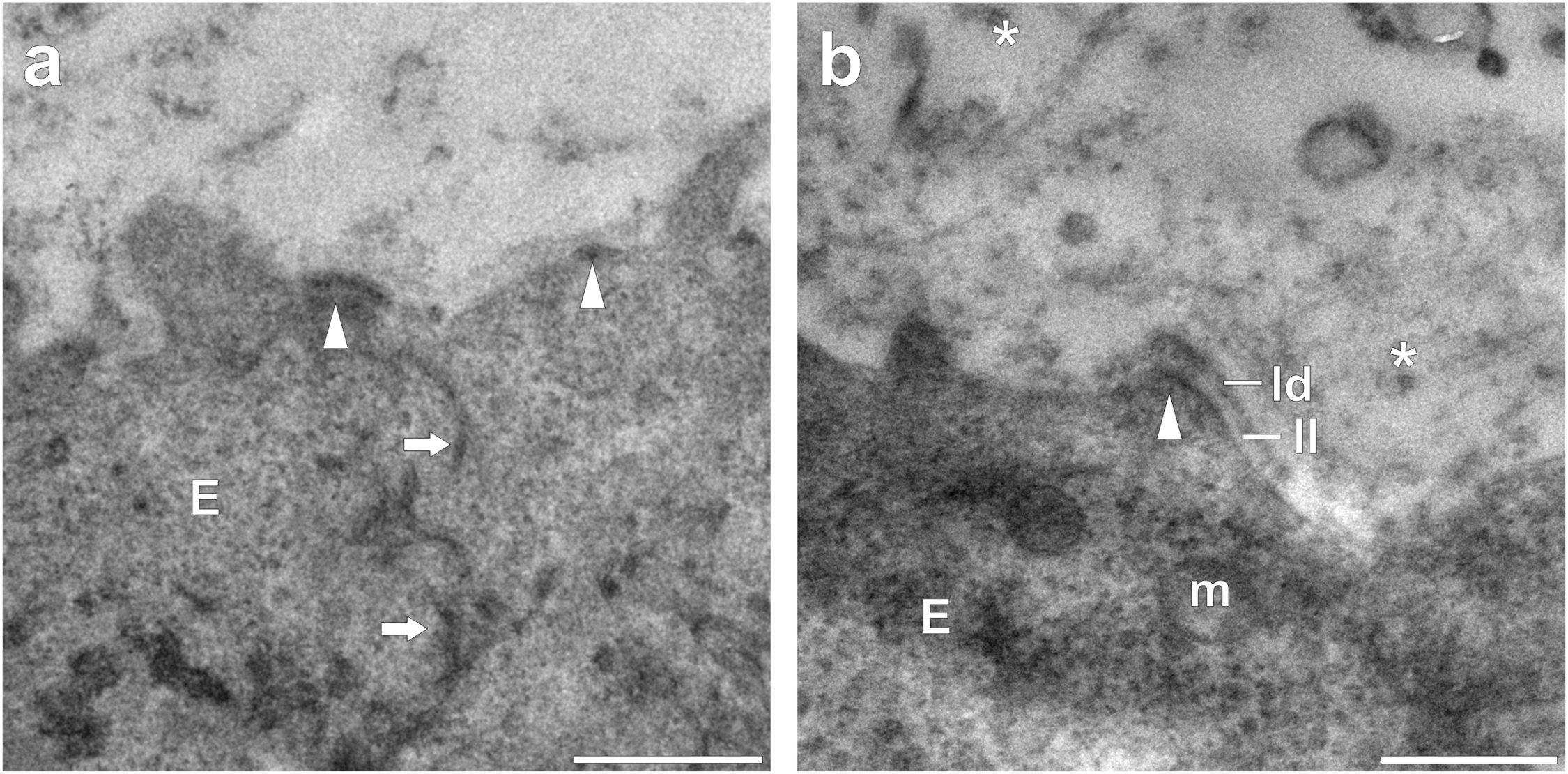

Supplement: S1 Fig — Dermoepithelial junction as present in samples of human-skin-equivalent organoids generated from donor cells of patients with recessive dystrophic epidermolysis bullosa (RDEB). Labelling. White asterisks: transversally sectioned collagen I/III fibrils in extracellular matrix, white arrows: keratin fibres, white arrowheads: hemidesmosomes, E: endothelial cell, ld and ll: lamina densa and lamina lucida of basement membrane, respectively, m: mitochondrion. (a) E-PTA staining accentuates keratin fibres inside the epidermal cells and hemidesmosomes connecting to an incompletely developed basal lamina. As characteristic for RDEB, collagen VII anchoring fibrils are missing, and no collagen I/III fibrils can be found in the vicinity of the epithelial cell. (b) Motif similar to that of (a) but showing a more conspicuous basal lamina, with the lamina lucida and lamina densa sublayers being clearly visible at the site of the hemidesmosome. Again, no collagen VII anchoring fibrils are discernible, and only a few collagen I/III fibrils are present in the matrix close to the cells. (TIF) [file pone.0339342.s002.TIF]
